# Supplementary figures and images for: Immune Prophylaxis Targeting the Respiratory Syncytial Virus (RSV) G Protein
Source: Viruses. 2023 Apr 27;15(5):1067. doi: 10.3390/v15051067 (PMC10221658; doi:10.3390/v15051067)

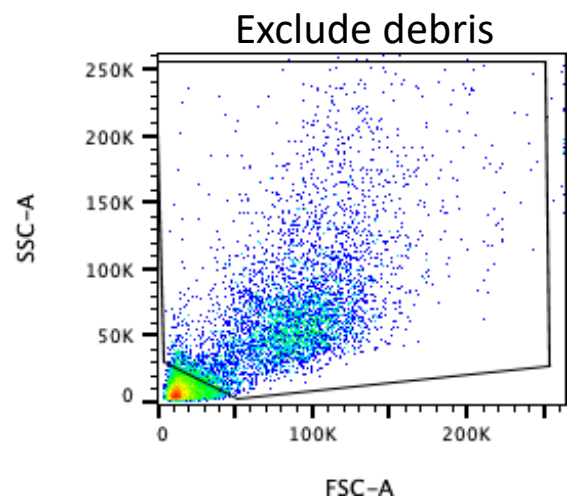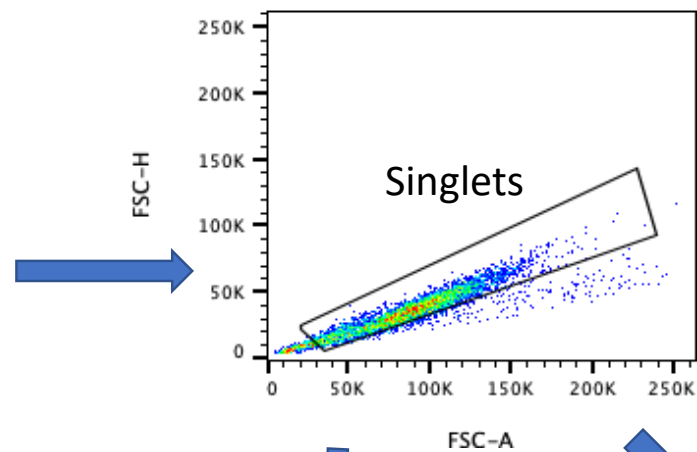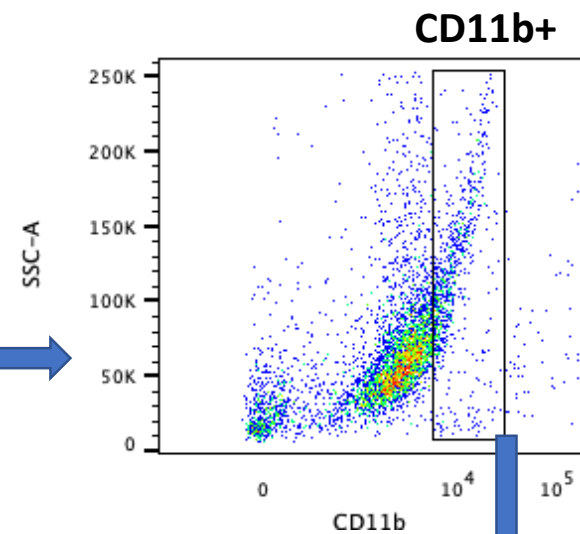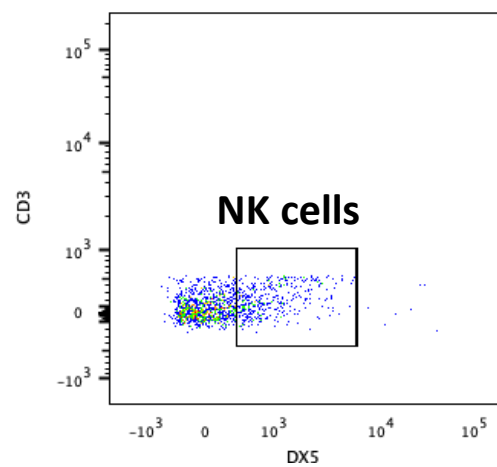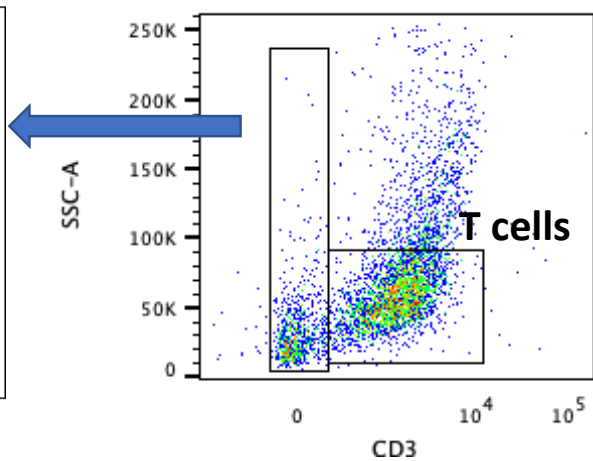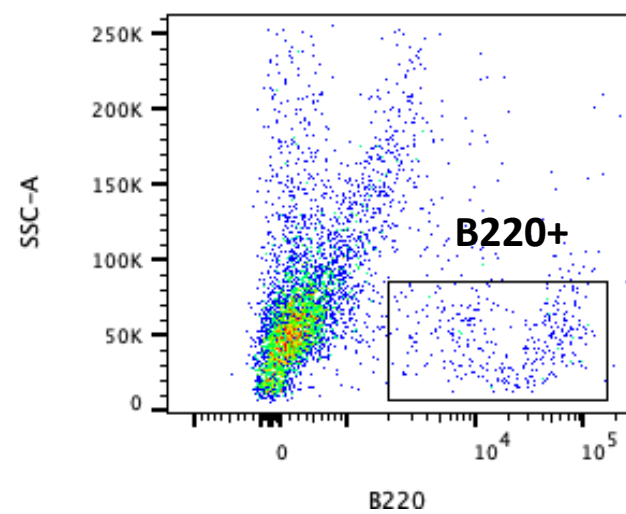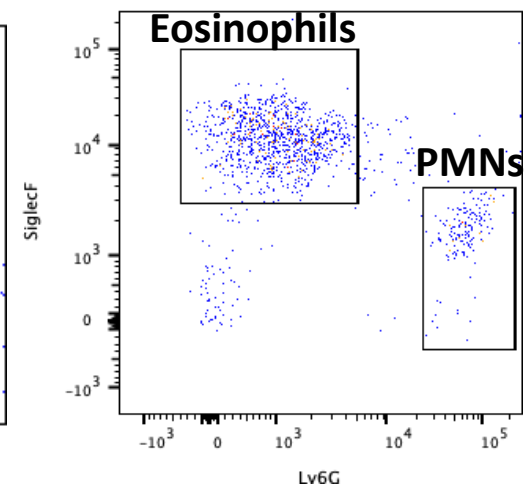

Supplement: Supplementary file 1 [file viruses-15-01067-s001.zip › viruses-2360715-supplementary.pdf]
